# Retrospective Evaluation of Hyperbilirubinemia in Cats and Dogs With Septic Peritonitis or Pyothorax

**DOI:** 10.1111/vec.70007

**Published:** 2025-07-29

**Authors:** Frederick John Benham‐Crosswell, Nieka Orire Arthur Payne, Lydia Joy Hjalmarsson, Karen R. Humm

**Affiliations:** ^1^ Department of Veterinary Clinical Sciences The Royal Veterinary College North Mymms Hertfordshire UK

**Keywords:** bilirubin, canine, feline, pyothorax, sepsis

## Abstract

**Objective:**

To characterize serum total bilirubin (TBil) value within 72 h of admission in cats and dogs with septic peritonitis or pyothorax and its relationship with outcome.

**Design:**

Cross‐sectional retrospective study.

**Setting:**

University teaching hospital.

**Animals:**

Sixteen cats with pyothorax, 28 cats with septic peritonitis, 49 dogs with pyothorax, and 86 dogs with septic peritonitis were included. Patients with evidence of hemolytic anemia, hepatic, or biliary disease were excluded.

**Methods:**

TBil within 72 h of admission, normalized to a range of 0–4.2 µmol/L (0–0.25 mg/dL) for dogs and 0–5.1 µmol/L (0–0.30 mg/dL) for cats (nTBil), length of hospitalization, and outcome (survived to discharge, euthanized, or cardiopulmonary arrest [CPA]) were recorded in each group of animals. The difference in nTBil between outcome groups was assessed using a Kruskal–Wallis test, and the difference in mortality between normobilirubinemic and hyperbilirubinemic patients was assessed using a *χ* ^2^ test.

**Results:**

The median (range) nTBil and frequency of hyperbilirubinemia were 6.3 (70.8) µmol/L (0.37 [4.14] mg/dL) and 82% in cats with septic peritonitis and 3.1 (120.3) µmol/L (0.18 [7.04] mg/dL) and 56% in cats with pyothorax. The median nTBil was significantly higher in cats that were euthanized or had CPA compared with survivors. Mortality was significantly higher in hyperbilirubinemic cats (48%) compared with normobilirubinemic cats (9%). There was no significant difference in median nTBil between dogs that survived, were euthanized, or had CPA. Mortality was significantly higher in hyperbilirubinemic dogs (45%) compared with normobilirubinemic dogs (28%).

**Conclusion:**

Hyperbilirubinemia is common in cats and dogs with pyothorax and septic peritonitis and is associated with an increased mortality.

**Abbreviations**
APPLE: Acute Patient Physiologic and Laboratory Evaluation
CPA: cardiopulmonary arrest
nTBil: normalized total bilirubin concentration: first bilirubin measurement taken and within 72 h of admission
RI: reference interval
SIRS: systemic inflammatory response syndrome
SOFA: sequential organ failure assessment
TBil: total bilirubin concentration

## 1 Introduction

Sepsis in people is defined as life‐threatening organ dysfunction resulting from a dysregulated host response to infection [1]. Despite a recent position statement [2], there is still no consensus definition for sepsis in cats and dogs [3]. Mortality is reported to be 20%–70% in cats and dogs [3, 4]. Many biomarkers and scoring systems have been investigated as diagnostic and prognostic factors in both human and veterinary patients with sepsis. Increased sequential organ failure assessment (SOFA) and quick SOFA scores, adapted from human medicine, are associated with increased mortality in critically ill dogs, including those diagnosed with sepsis [5, 6]. The SOFA scoring system requires an assessment of hepatic function, which is often determined by measurement of serum total bilirubin concentration (TBil) [5]. TBil is also included in the full Acute Patient Physiologic and Laboratory Evaluation (APPLE) canine score (designed for illness stratification) [7]. In people with sepsis, high serum bilirubin concentration within 72 h of admission to the ICU is associated with increased mortality [8]. However, in one study of dogs looking at multiple parameters related to different organs in sepsis, TBil was not independently associated with mortality [9].

Bilirubin is a hemoglobin breakdown byproduct that is produced in reticuloendothelial cells and released into the bloodstream. In the liver, bilirubin is conjugated to glucuronic acid and excreted through the biliary system. TBil is a measurement of both conjugated and unconjugated bilirubin present in the blood. It may be increased due to excessive RBC breakdown, hepatic dysfunction, or biliary system obstruction. Increased TBil is common in sepsis and is thought to result primarily from increased cholestasis, but oxidative or hypoxic damage to hepatocytes driven by the dysregulated, proinflammatory state of the immune system also contributes [10]. Cholestasis is caused by damage to the hepatocyte cytoskeleton and bile salt pumps and transporters [11]. The liver has a large population of macrophages (Kupffer cells), which are stimulated by bacterial endotoxin, further driving the proinflammatory state and cholestasis [12]. At physiological concentrations, bilirubin is an antioxidant and anti‐inflammatory immunomodulant. However, at high concentrations, it uncouples oxidative phosphorylation, inhibits DNA and protein synthesis, and inhibits neutrophil phagocytosis [13].

Serum bilirubin concentration can be increased in septic patients and may be prognostic. However, the variation in TBil in septic dogs and cats has not been described. Given the lack of a consensus definition of sepsis in veterinary medicine, it is difficult to study a veterinary population that is universally recognized as septic. This study aimed to describe TBil in cats and dogs with pyothorax or septic peritonitis, both of which are conditions recognized to cause sepsis. Secondary aims were to determine whether TBil had prognostic value and whether TBil within 72 h of admission was correlated to length of hospitalization in survivors.

## 2 Materials and Methods

The clinical records of a referral hospital were searched between January 1, 2009, and December 31, 2020, using the terms “pyothorax,” “septic abdomen,” and “septic peritonitis.” Dogs and cats with a diagnosis of septic peritonitis or pyothorax were included. Patients were excluded if serum bilirubin concentration was not recorded within 72 h of admission, if septic peritonitis or pyothorax developed only after admission, or if a diagnosis or clinical suspicion of hemolytic anemia, primary hepatic, or cholestatic disease was present in the medical records, either before referral or after admission. All patients had CBC, serum biochemistry panel, and point‐of‐care ultrasound examination performed. Septic peritonitis or pyothorax was confirmed with cytology, culture, or histopathology in all cases. The signalment, first TBil measurement recorded (which had to be within 72 h of admission), length of hospitalization, and outcome (discharge, cardiopulmonary arrest [CPA], or euthanasia) were recorded. TBil within 72 h of admission was measured using 1 of 2 biochemical analyzers^a^^,^ b or a blood gas analyzer.^c^ Hemolyzed samples were excluded. Because three different machines, each with a different reference interval (RI) for dogs and cats were used, TBil values were normalized (nTBil) to correspond with the RI of the most commonly used biochemical analyzer^b^ using the formula [14]:

$$Y=\left(\frac{X-X_{\min}}{X_{range}}\right)n$$

where *X* is the original recorded bilirubin concentration, *X*_min_ is the lower limit of the original RI, *X*_range_ is the difference between the upper and lower limits of the original RI, and *n* is the upper limit of the new RI, which was 0–5.1 µmol/L (0–0.30 mg/dL) for cats and 0–4.2 µmol/L (0–0.25 mg/dL) for dogs.

Graphical and statistical analyses were carried out using a statistical computing package.^d^ A Shapiro–Wilks test was used to assess normality of nTBil for each species, condition, and outcome. Normally distributed data were reported as mean (± SD) and non‐normal data as median with range. Cats and dogs were analyzed separately due to differences in species. A Kruskal–Wallis rank‐sum test was used to test the difference in nTBil values between groups of dogs or cats with differing outcomes. This test was carried out once to determine the difference between survival, euthanasia, and CPA outcomes, and once with only survival and CPA outcomes. Spearman's rank correlation was used to test for correlation between nTBil within 72 h of admission and length of hospitalization in surviving patients. Survival to discharge percentages were calculated for each species and condition. After this analysis, cases were stratified into five cohorts based on their nTBil value: within RI, above the RI but within twice the upper limit; twice above but within four times the upper limit; ≥ 4 times above but within eight times the upper limit; and ≥ 8 times the upper limit. A *χ* ^2^ test assessed whether mortality differed between hyperbilirubinemic and normobilirubinemic dogs or cats. A second *χ* ^2^ test assessed differences in mortality between stratification groups.

## 3 Results

**FIGURE 1:**
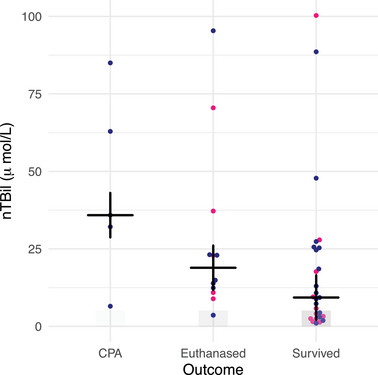
Dot plot of normalized total bilirubin concentration (µmol/L) for 44 cats with pyothorax or septic peritonitis that survived, were euthanized, or experienced cardiopulmonary arrest. Pink dots represent cats with pyothorax and blue dots represent cats with septic peritonitis. The gray‐shaded area of the y‐axis indicates the normal nTBil interval (0–5.1 µmol/L [0–0.30 mg/dL]). The cross indicates the median value for each group. *p* = 0.031. Abbreviation: nTBil, normalized total bilirubin.

**FIGURE 2:**
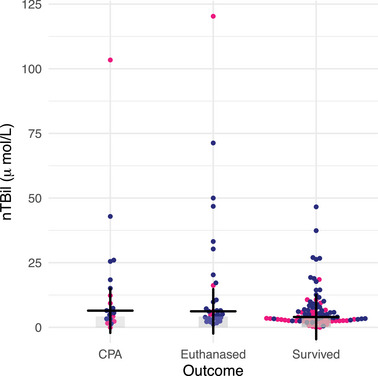
Dot plot of normalized total bilirubin concentration (µmol/L) for 135 dogs with pyothorax or septic peritonitis that survived, were euthanized, or experienced cardiopulmonary arrest. Pink dots represent dogs with pyothorax and blue dots represent dogs with septic peritonitis. The gray area of the y‐axis indicates the normal nTBil interval (0–4.2 µmol/L [0–0.25 mg/dL]). The cross indicates the median value for each group. *p* = 0.092. Abbreviation: nTBil, normalized total bilirubin.

The study included 179 patients: 16 cats with pyothorax, 28 cats with septic peritonitis, 49 dogs with pyothorax, and 86 dogs with septic peritonitis. The mean age was 5.0 ± 3.4 years in cats with pyothorax, 6.6 ± 3.2 years in cats with septic peritonitis, 4.6 ± 3.3 years in dogs with pyothorax, and 6.1 ± 3.7 years in dogs with septic peritonitis. Figures 1 and 2 show the variation in nTBil among species, outcome, and underlying disease process. The nTBil values within 72 h of admission for each condition are shown in Table 1 for cats and Table 2 for dogs.

**TABLE 1:** Median, range, and maximum normalized total bilirubin concentration (µmol/L and mg/dL) in 44 cats with pyothorax or septic peritonitis reported by number of animals for each condition and outcome.

Condition	Outcome	Number of cases	Median nTBil (µmol/L)	Range (µmol/L)	Maximum nTBil value (µmol/L)	Median nTBil (mg/dL)	Range (mg/dL)	Maximum nTBil value (mg/dL)
Pyothorax	Any	16	9.2	98.9	100.3	0.54	5.78	5.87
Pyothorax	Survived	11	4.1	98.9	100.3	0.24	5.78	5.87
Pyothorax	Euthanized	5	22.9	61.6	70.5	1.34	3.60	4.12
Pyothorax	CPA	0	NA	NA	NA	NA	NA	NA
Septic peritonitis	Any	28	16.7	94.4	95.4	0.98	5.52	5.58
Septic peritonitis	Survived	16	11.9	87.6	88.6	0.70	5.12	5.18
Septic peritonitis	Euthanized	7	14.9	91.8	95.4	0.87	5.37	5.58
Septic peritonitis	CPA	5	35.9	78.5	85.0	2.10	4.59	4.97
Either condition (all cats)	Any	44	13.5	99.3	100.3	0.79	5.81	5.87
Either condition	Survived	27	9.3	99.3	100.3	0.54	5.81	5.87
Either condition	Euthanized	12	18.9	91.8	95.4	1.11	5.37	5.58
Either condition	CPA	5	35.9	78.5	85.0	2.10	4.59	4.97

Abbreviations: CPA, cardiopulmonary arrest; nTBil, normalized total bilirubin concentration.

**TABLE 2:** Median, range, and maximum normalized total bilirubin concentration (µmol/L and mg/dL) in 135 dogs with pyothorax or septic peritonitis reported by number of animals for each condition and outcome.

Condition	Outcome	Number of cases	Median nTBil (µmol/L)	Range (µmol/L)	Maximum nTBil value (µmol/L)	Median nTBil (mg/dL)	Range (mg/dL)	Maximum nTBil value (mg/dL)
Pyothorax	Any	49	3.1	120.3	120.3	0.18	7.04	7.04
Pyothorax	Survived	38	3.0	18.5	18.5	0.18	1.08	1.08
Pyothorax	Euthanized	4	11.2	115.4	120.3	0.65	6.75	7.04
Pyothorax	CPA	7	4.2	103.4	103.4	0.25	6.05	6.05
Septic peritonitis	Any	86	6.3	70.8	71.3	0.37	4.14	4.17
Septic peritonitis	Survived	47	6.1	46.1	46.6	0.36	2.70	2.73
Septic peritonitis	Euthanized	26	5.7	70.1	71.3	0.33	4.10	4.17
Septic peritonitis	CPA	13	6.5	41.5	42.9	0.38	2.43	2.51
Either condition (all dogs)	Any	135	5.0	120.3	120.3	0.29	7.04	7.04
Either condition	Survived	85	4.0	46.6	46.6	0.23	2.73	2.73
Either condition	Euthanized	20	6.2	119.1	120.3	0.36	6.96	120.3
Either condition	CPA	5	6.5	65.6	70.0	0.38	3.84	4.09

Abbreviations: CPA, cardiopulmonary arrest; nTBil, normalized total bilirubin concentration.

The median duration of hospitalization of cats that survived to discharge was 7.0 days (range, 2.5), and there was a weak but significant correlation between nTBil and hospitalization length (*r*_s_ = 0.40, *p* = 0.039). The median duration of hospitalization of dogs that survived to discharge was 7.0 days (range, 2.5), and there was no correlation found between nTBil and hospitalization length (*r*_s_ = 0.12, *p* = 0.26).

Overall, 61% of cats survived to discharge, 27% were euthanized, and 12% experienced CPA. Sixty‐nine percent of cats with pyothorax survived, 31% were euthanized, and none experienced CPA. Fifty‐seven percent of cats with septic peritonitis survived, 25% were euthanized, and 18% experienced CPA. Sixty‐three percent (10/16) of cats with pyothorax and 82% (23/28) of cats with septic peritonitis were hyperbilirubinemic. The median nTBil was significantly different between outcome groups (*p* = 0.039). It was highest in cats that experienced CPA, lower in cats that were euthanized, and lowest in cats that survived to discharge. When euthanized cats were excluded, those that experienced CPA had a significantly higher nTBil than those that survived (*p* = 0.031; Table 1).

Mortality was 48% in hyperbilirubinemic cats, significantly higher than the 9% mortality in normobilirubinemic cats (*p* = 0.020). However, as the nTBil cohort increased, no significant increase in mortality was seen (*p* = 0.14). Mortality was 9% in normobilirubinemic cats, 29% in cats with nTBil between once and twice the RI upper limit, and 54% for cats with nTBil with more than twice the RI upper limit. Mortality was similar for all nTBil cohorts beyond twice the RI (Table 3).

**TABLE 3:** Total number of cats, number of survivors, mortality, and proportion of all cases for each cohort in 44 total cats with pyothorax or septic peritonitis based on normalized total bilirubin concentration (µmol/L and mg/dL; *p* = 0.13).

Bilirubin value	nTBil (µmol/L)	nTBil (mg/dL)	Number of survivors	Number of patients	Mortality (%)	Proportion of all cases in this group (%)
> 8 × RI	> 40.8	> 2.4	4	8	50	18
4–8 × RI	20.4–40.8	1.2–2.4	5	11	55	25
2–4 × RI	10.2–20.4	0.6–1.2	3	7	57	16
1–2 × RI	5.1–10.2	0.3–0.6	5	7	29	16
Within RI	0–5.1	0–0.3	10	11	9	25
Total	—	—	27	44	40	100

Abbreviations: nTBil, normalized total bilirubin concentration; RI, reference interval.

Overall, 63% of dogs survived to discharge, 22% were euthanized, and 15% experienced CPA. Seventy‐eight percent of dogs with pyothorax survived, 8% were euthanized, and 14% experienced CPA. Fifty‐five percent of dogs with septic peritonitis survived, 30% were euthanized, and 15% experienced CPA. Thirty‐four percent (17/49) of dogs with pyothorax and 60% (57/86) of dogs with septic peritonitis were hyperbilirubinemic. There was no difference in the median nTBil between dogs that had CPA, were euthanized, or survived to discharge (*p* = 0.092). When euthanized dogs were excluded, there was still no difference between dogs that had CPA and dogs that survived (*p* = 0.16; Table 2).

Mortality was greater in hyperbilirubinemic dogs (45%) than in normobilirubinemic dogs (28%; *p =* 0.045). There was no difference in mortality between nTBil cohorts (*p* = 0.081). Mortality was 28% in normobilirubinemic dogs and 75% in dogs with nTBil ≥ 8 times above the RI upper limit (Tables 4 and 5)

**TABLE 4:** Total number of dogs, number of survivors, mortality, and proportion of all cases for each cohort in 135 total dogs with pyothorax or septic peritonitis based on normalized total bilirubin concentration (µmol/L and mg/dL; *p* = 0.081).

Bilirubin value	nTBil (µmol/L)	nTBil (mg/dL)	Number of survivors	Number of patients	Mortality (%)	Proportion of all cases in this group (%)
> 8 × RI	> 33.6	> 1.96	2	8	75	6
4–8 × RI	16.8–33.6	0.98–1.96	7	14	50	10
2–4 × RI	8.4–16.8	0.49–0.98	11	17	35	13
1–2 × RI	4.2–8.4	0.25–0.49	21	35	40	26
Within RI	0–4.2	0–0.25	44	61	28	45
Total	—	—	85	135	46	100

Abbreviations: nTBIL, normalized total bilirubin concentration; RI, reference interval.

**TABLE 5:** *p*‐values for each statistical test comparing normalized total bilirubin concentration (nTBil) in 44 cats and 135 dogs with either pyothorax or septic peritonitis.

Statistical test	Cats	Dogs
nTBil vs. length of hospitalization (Spearman's rank)	**0.039**	0.26
Median nTBil vs. outcome (Kruskal–Wallis)	**0.039**	0.092
Median nTBil vs. outcome, excluding euthanized (Kruskal–Wallis)	**0.031**	0.16
Hyperbilirubinemic vs. survival (*χ* ^2^)	**0.020**	**0.045**
nTBil stratification vs. survival (*χ* ^2^)	0.13	0.081

*Note*: Significant values are shown in bold.

Abbreviation: nTBil, normalized total bilirubin concentration.

## 4 Discussion

The current study aimed to determine the frequency of hyperbilirubinemia and the concentrations of nTBil seen in cats and dogs with pyothorax and septic peritonitis. The concentration of nTBil was highly variable among patients; however, the majority were hyperbilirubinemic. Hyperbilirubinemia was more frequent in cats than in dogs. There is no universally accepted veterinary definition of sepsis, but both pyothorax and septic peritonitis are conditions that can cause sepsis in dogs and cats. Therefore, the hyperbilirubinemia in these patients is thought to have been secondary to sepsis, and the frequency and severity of hyperbilirubinemia reported may help understand the expected TBil in different septic canine and feline populations.

In one human study of patients classified as having severe sepsis, only 20% were hyperbilirubinemic [8] compared with 75% of the cats and 55% dogs in our study. Only 5% of these human patients had TBil twice above the upper end of the RI, which was 17.1 µmol/L (1.0 mg/dL), compared with 59% of the cats and 39% of the dogs in the current study, where the upper end of the RI was 5.1 µmol/L (0.30 mg/dL) for cats and 4.2 µmol/L (0.25 mg/dL) for dogs. Other human studies have reported the prevalence of hyperbilirubinemia within septic adult patients between 0.6% and 54% [10]. In a canine septic peritonitis study, 14% of cases had liver involvement, defined as 2 of the following: TBil > 42 µmol/L (2.5 mg/dL, 10 times the upper RI limit in the current study), alanine aminotransferase > twice the RI upper limit, prothrombin time or activated partial thromboplastin time > 1.5 times the RI [11]. This is comparable to the 6% of dogs and 18% of cats in the current study with nTBil ≥ 8 times the RI upper limit.

Mortality in normobilirubinemic cats was notably low at 9% (overall feline mortality was 40%), and mortality increased with hyperbilirubinemia. The same association was present in dogs, although the difference in mortality between normobilirubinemic and hyperbilirubinemic dogs was smaller than in cats. A more powerful study with a larger sample size would be beneficial to determine if this is a replicable finding and assess how mortality varies between different TBil value cohorts. We suspect that, in cats, the mortality suddenly increases at around twice the nTBil reference range, whereas in dogs it steadily increases with increased nTBil, but we do not have sufficient data to investigate this. In both species, such data could help optimize scoring systems, such as APPLE [7] and SOFA [5], which use TBil and other parameters to stratify septic patients by illness severity. Although we found an association between hyperbilirubinemia and mortality, another study [9] found no association, and it is likely that TBil's predictive value on outcome is weak; TBil may not have a role in future canine sepsis scoring systems.

Pyothorax and septic peritonitis were chosen as conditions to study because sample collection to demonstrate an infectious organism via cytology, culture, or histopathology is routine, and there are life‐threatening systemic consequences to the infection. The definition of sepsis in people, “life‐threatening organ dysfunction caused by a dysregulated host response to infection,” [1] is not standardized in veterinary patients; therefore, it is difficult to state a population is definitively septic. This patient population had a bacterial cause of systemic illness, and all cats and dogs were hospitalized in an ICU. The overall mortality of 37% is consistent with human sepsis. However, a more rigorous definition of life‐threatening organ dysfunction, such as SOFA or APPLE scores, could have been used to define this population as septic. TBil is used to help identify people with sepsis as a marker of hepatic dysfunction. It is not clear what TBil should be used to define liver dysfunction in cats and dogs, and the TBil used in different studies varies greatly. An accepted TBil that indicates liver dysfunction and a clear definition of veterinary sepsis with accepted guidelines for life‐threatening organ dysfunction in septic veterinary patients are both needed.

Sepsis can arise from other conditions, such as pneumonia, prostatitis, and pyometra; however, definitive sampling for demonstration of an infectious organism is more variable, and it can be more difficult to identify which of these patients are septic, particularly when performing a retrospective study. It would be interesting to know if TBil is increased as often or to the same degree with these other causes of sepsis or indeed in noninfectious cases of severe inflammatory response syndrome (SIRS). In people, TBil is significantly higher in patients with sepsis patients compared with patients with noninfectious SIRS (although it can be elevated in patients with noninfectious SIRS) [15]. This may be due to bacterial endotoxin triggering the Kupffer cells of the liver.

This study was limited by a small sample size, particularly with the population separated by species with two different disease processes. This was especially true for the extremely hyperbilirubinemic cohort, in which there were only eight cats and eight dogs. A study with a larger sample size is needed that can separately analyze pyothorax and septic peritonitis cases. Another limitation was that the timing of nTBil measurement was variable relative to the initiation of the disease process because it was measured within 72 h of presentation to a referral hospital. In most cases, some treatment was initiated at a referring clinic, making it difficult to know when the disease process began. It would have been interesting to note the peak nTBil for each patient and how it may have changed with treatment but, given the varying sampling times, it was not possible to accurately report these values.

It is also important to note that, although attempts were made to exclude patients with hepatobiliary disease not related to sepsis, the retrospective nature of the study prevents such control over baseline characteristics. Although some patients had thorough investigation of the hepatobiliary system with diagnostic imaging or exploratory laparotomy (including almost all patients with septic peritonitis), this was not carried out in all patients. Another limitation was the inclusion of patients that were euthanized, since a patient's TBil may influence clinicians’ recommendations regarding clinical care including euthanasia. However, a difference was found between nTBil in surviving cats versus those that died naturally, suggesting the association between TBil and survival may be accurate. Additionally, only a small population of dogs and cats with two specific conditions at a single institution were studied. Finally, the diversity in species and underlying causes of disease, as well as the lack of a consensus veterinary sepsis definition, make it impossible to state that all these cats and dogs were septic.

In conclusion, hyperbilirubinemia is common in cats and dogs with septic peritonitis or pyothorax. In cats, mortality was 9% in normobilirubinemic patients and 54% in cats with nTBil > double the RI upper limit, and in dogs, mortality was 28% in normobilirubinemic patients and 75% in patients with nTBil > 8 times the RI upper limit. Hyperbilirubinemia was associated with increased mortality in both species. Recognizing the possible severity of sepsis‐associated hyperbilirubinemia is important to prevent unnecessary investigations into other possible causes of the increased TBil. There is insufficient evidence to use TBil as a prognostic indicator given small population and the wide variation seen in both survivors and nonsurvivors.

## Author Contributions

**Frederick John Benham‐Crosswell**: Conceptualization; data curation; formal analysis; investigation; methodology; project administration; validation; visualization; writing – original draft. **Nieka Orire Arthur Payne**: Conceptualization; data curation; formal analysis; investigation; methodology; visualization; writing – original draft. **Lydia Joy Hjalmarsson**: Conceptualization; data curation; formal analysis; investigation; methodology; validation; visualization; writing – original draft. **Karen R. Humm**: Conceptualization; formal analysis; investigation; methodology; project administration; supervision; writing – review & editing.

## Conflicts of Interest

The authors declare no conflicts of interest.

## Endnotes

a Ilab600, Shimadzu UK, Milton Keynes, UK.

b Au680 Clinical chemistry analyzer, Beckman Coulter UK, High Wycombe, UK.

c Stat profile prime plus, Nova biomedical UK, London, UK.

d R studio 2017, R Foundation for Statistical Computing, Vienna, Austria.
